# Weighted single-step genomic BLUP improves accuracy of genomic breeding values for protein content in French dairy goats: a quantitative trait influenced by a major gene

**DOI:** 10.1186/s12711-018-0400-3

**Published:** 2018-06-15

**Authors:** Marc Teissier, Hélène Larroque, Christèle Robert-Granié

**Affiliations:** GenPhySE, INRA, INPT, ENVT, Université de Toulouse, 31326 Castanet-Tolosan, France

## Abstract

**Background:**

In 2017, genomic selection was implemented in French dairy goats using the single-step genomic best linear unbiased prediction (ssGBLUP) method, which assumes that all single nucleotide polymorphisms explain the same fraction of genetic variance. However, ssGBLUP is not suitable for protein content, which is controlled by a major gene, i.e. *α*_*s1*_ *casein*. This gene explains about 40% of the genetic variation in protein content. In this study, we evaluated the accuracy of genomic prediction using different genomic methods to include the effect of the *α*_*s1*_ *casein* gene.

**Methods:**

Genomic evaluation for protein content was performed with data from the official genetic evaluation on 2955 animals genotyped with the Illumina goat SNP50 BeadChip, 7202 animals genotyped at the *α*_*s1*_ *casein* gene and 6,767,490 phenotyped females. Pedigree-based BLUP was compared with regular unweighted ssGBLUP and with three weighted ssGBLUP methods (WssGBLUP, WssGBLUP_Max_ and WssGBLUP_Sum_), which give weights to SNPs according to their effect on protein content. Two other methods were also used: trait-specific marker-derived relationship matrix (TABLUP) using pre-selected SNPs associated with protein content and gene content based on a multiple-trait genomic model that includes *α*_*s1*_ *casein* genotypes. We estimated accuracies of predicted genomic estimated breeding values (GEBV) in two populations of goats (Alpine and Saanen).

**Results:**

Accuracies of GEBV with ssGBLUP improved by + 5 to + 7 percent points over accuracies from the pedigree-based BLUP model. With the WssGBLUP methods, SNPs that are located close to the *α*_*s1*_ *casein* gene had the biggest weights and contributed substantially to the capture of signals from quantitative trait loci. Improvement in accuracy of genomic predictions using the three weighted ssGBLUP methods delivered up to + 6 percent points of accuracy over ssGBLUP. A similar accuracy was obtained for ssGBLUP and TABLUP considering the 20,000 most important SNPs. Incorporating information on the *α*_*s1*_ *casein* genotypes based on the gene content method gave similar results as ssGBLUP.

**Conclusions:**

The three weighted ssGBLUP methods were efficient for detecting SNPs associated with protein content and for a better prediction of genomic breeding values than ssGBLUP. They also combined fast computing, simplicity and required ssGBLUP to be run only twice.

## Background

The availability of molecular data has enabled the development and commercial application of genomic selection in various livestock species, such as dairy cattle [1, 2], dairy sheep [3, 4], meat sheep [5, 6] and dairy goats [7–9]. Meuwissen et al. [10] proposed genomic prediction of animals based on dense single nucleotide polymorphism (SNP) maps, by deriving the effects of SNPs from a reference population, for which animals are both phenotyped and genotyped. Genomic estimated breeding values (GEBV) of selection candidates (i.e., usually young individuals with genotypes but without phenotypes) can be estimated by summing up the effects of the SNP alleles carried by each animal.

Methods such as genomic best linear unbiased prediction (GBLUP) [11–15], are used to predict GEBV by replacing the pedigree relationship matrix used for pedigree-based BLUP with a realized genomic relationship matrix. The GBLUP method was further improved with single-step GBLUP (ssGBLUP) [12], which uses simultaneously all phenotypic, pedigree and genotypic information, including phenotypic information on non-genotyped individuals. Therefore, in ssGBLUP, the relationship between each pair of animals (genotyped and non-genotyped) is estimated with a relationship matrix that combines pedigree and genotype information. Several studies have reported that the accuracy of genomic prediction obtained with these methods is higher than with genetic evaluation using pedigree-based BLUP [16–18]. However, the accuracy obtained from genomic information depends on several parameters including reference population size [19, 20], extent of linkage disequilibrium (LD), heritability of the trait [20, 21], relationship between training and validation populations [10] and the genetic architecture of the trait, which relates to the relative size of allele substitution effects at quantitative trait loci (QTL) [10, 22].

The GBLUP and ssGBLUP methods usually assume that each SNP follows the same distribution [11, 12, 16, 23–25], thus, all SNPs have the same variance and the same weight for SNP variance. However, different genomic evaluation methods have been developed to allow the variance of the effect of SNPs to differ between SNPs. A priori information can be used to modify the distribution of SNP effects. Giving more variance to some SNPs allows these methods to take the presence of major genes or QTL that affect the trait of interest into account. For instance, various Bayesian methods, which estimate the effect of SNPs from animals that are both genotyped and phenotyped, have been proposed [10, 26–28]. The main difference between these Bayesian methods lies in the definition of an a priori distribution of the effects of SNPs. SNPs can be attributed to different distribution classes, which explain different parts of the total genetic variance, with one class possibly containing the SNPs that have no effect on the trait. Because animals need to be phenotyped and genotyped to apply Bayesian methods, phenotypes from non-genotyped animals cannot be included. In dairy breeding programs, genotypes are mainly determined on the males whereas phenotypes come from the females. Thus, daughter yield deviations (DYD) or de-regressed proofs are calculated to obtain pseudo-phenotypes for the males. However, multi-step methods may create bias in genomic predictions [29].

Other methods based on the ssGBLUP framework such as weighted ssGBLUP (WssGBLUP) or on the trait-specific marker-derived relationship matrix (TABLUP) have been proposed [30]. WssGBLUP is an extension of ssGBLUP in which weights for SNP variances are used when forming the genomic relationship matrix [12]. WssGBLUP can set more weight to SNPs that are in high LD with a causal mutation or associated with QTL with a relatively large effect. These weights are estimated from the variance explained by each SNP as presented by Wang et al. [23]. The weighting of SNP variances was also investigated by Zhang et al. [24] who proposed to use the same weight for SNPs that are within a defined window along the genome. The TABLUP method proposes to construct the genomic relationship matrix based on genotypes from a subset of pre-selected SNPs. Selection of SNPs can be performed after GWAS analysis or based on weights that are estimated with WssGBLUP. The selected SNPs are then equally weighted for the analyses [30]. Furthermore, an alternative to the previous methods is the gene content method proposed by Gengler et al. [31], which is based on a multiple trait model and considers the gene content for specific genotypes as a new trait. This method can combine information from SNPs and genotypes for a causal mutation [31, 32]. The number of alleles carried by each animal is considered as a second trait correlated to the quantitative trait. Then, the causal mutation is integrated directly in the ssGBLUP multiple-trait model. Its advantage is that it can be extended to multi-allelic genes and used when genotypes for a causal mutation are missing [33].

In French dairy goats, the first step towards genomic selection for milk production traits, udder type traits and somatic cell score was taken by Carillier-Jacquin et al. [8, 9] for French Alpine and Saanen dairy goat breeds. Carillier-Jacquin et al. [8, 9] compared ssGBLUP and other methods of genomic evaluation that require several steps (GBLUP or Bayesian methods). GBLUP and Bayesian methods usually use performances based on pseudo-phenotypes (DYD) whereas ssGBLUP is based on female performance. These authors found that ssGBLUP gave more accurate predictions of the genetic merit of selection candidates than the previous official genetic evaluation that did not use genomic information, or the use of multi-step genomic methods. However, the increase in accuracy due to using genomic information was not expected to be high because the reference population was small.

Currently, the next step in the genomic evaluation of French dairy goats is to investigate better ways to use genotyping information to improve the accuracy of genomic evaluation. One possibility is to take prior knowledge about major genes into account. Several major genes have been identified, such as *DGAT1* for fat content [34] and *α*_*s1*_ *casein* for protein content [35]. For protein content, Carillier-Jacquin et al. [33] reported that the genetic variance explained by the *α*_*s1*_ *casein* gene reached 38% in the Saanen and 43% in the Alpine breed. The caprine *α*_*s1*_ *casein* gene has six alleles ($A$, $B$, $C$, $E$, $F$ and $O$) that have been identified in the French dairy goat population. Allele $A$ is predominant in the Alpine breed, whereas alleles $A$, $E$ and $F$ are the most frequent in the Saanen breed [33]. Carillier-Jacquin et al. [33] showed that integrating the *α*_*s1*_ *casein* gene for protein content with the gene content method improved the accuracy of genomic evaluation (+ 8 to 14% for Alpine and Saanen populations) compared with ssGBLUP.

In this study, our aim was to investigate different methods of genomic prediction that estimate and integrate the fact that chromosomal regions are strongly associated with a trait. Protein content in French dairy goats was analyzed by applying WssGBLUP, two alternatives of the WssGBLUP method, the TABLUP method and the gene content method. These methods were compared with pedigree-based BLUP and ssGBLUP based on the accuracies of predicted breeding values.

## Methods

### Animals, phenotypes and genotypes

The dataset used in this study was provided by the French national milk records system and included animals from the two main French dairy goat breeds, Alpine and Saanen. Phenotypes for protein content, pedigree data, genotypes and environmental fixed effects used in the ssGBLUP method were obtained from the official genetic evaluation of January 2016 [36]. Analyses were performed with a multi-breed dataset (Alpine and Saanen animals combined) and in two separate within-breed analyses.

The trait analyzed was protein content (g/kg) with measurements from 6,767,490 lactations and 2,458,453 females recorded between 1980 and 2010. Descriptive statistics (animal and record numbers, minimum, mean, maximum, coefficient of variation) for each breed are in Table 1.

**Table 1:** Summary statistics on protein content (g/kg) in Alpine and Saanen breeds

Breed	Number of lactations	Number of females with phenotypes	Minimum^a^ (g/kg)	Mean^a^ (g/kg)	Maximum^a^ (g/kg)	CV
Alpine	3,844,071	1,392,399	10.47	30.42	54.81	0.11
Saanen	2,923,419	1,066,054	10.00	29.67	54.63	0.09

*CV* coefficient of variation

^a^Minimum, mean, maximum protein content

The pedigree consisted of 2,543,789 animals (1,449,991 Alpine and 1,093,798 Saanen). In addition, it was completed with 36 unknown parent groups. Unknown parent groups were defined for each breed and for animals born before 1975, and then for cohorts born in 2-year windows up to 2010.

Animals that were genotyped with the Illumina goat SNP50 BeadChip (50K SNP) [37] were also used in the analysis. Quality control (QC) for a dataset of 3347 genotyped animals (2020 Alpine and 1278 Saanen) and 53,347 SNPs was performed independently for each breed. SNPs with a minor allele frequency (MAF) lower than 1% and a call rate lower than 95% were removed. Hardy–Weinberg equilibrium was also tested and the associated Chi squared statistic was calculated for each SNP. SNPs with a Chi squared statistic higher than 24 were removed. Finally, animals with a SNP call rate lower than 99% were discarded from the analyses. After QC, 2955 (1749 Alpine and 1206 Saanen) animals and 46,849 SNPs remained for further analyses. Some SNPs within the *α*_*s1*_ *casein* gene were present on the 50 K SNP but since they did not pass QC, they were removed [33].

Genotypes for the *α*_*s1*_ *casein* gene were available for 3696 Alpine individuals (2154 males and 1542 females), and 3506 Saanen individuals (2049 males and 1457 females) born between 1982 and 2012. The *α*_*s1*_ *casein* gene is located on caprine chromosome 6 at 82 Mb and is multi-allelic in the French dairy goat population, with six different alleles ($A$, $B$, $C$, $E$, $F$ and $O$) and 19 genotypes detected among the 21 possibilities ($FO$ and $OO$ genotypes have never been detected in the French dairy goat population) [33]. Genotypes of animals with one missing allele were removed from the analysis. The estimated effects of the 19 *α*_*s1*_ *casein* genotypes on protein content were computed and reported previously [33]. Table 2 includes the number of animals (males and females for Alpine and Saanen breeds) used in this study with information on their *α*_*s1*_ *casein* and/or 50 K SNP genotypes.

**Table 2:** Number of animals with information on the *α*_*s1*_ *casein* genotype and/or 50 K SNP genotypes

Breed	Gender	Animals with 50 K SNP genotype	Animals with *α*_*s1*_ *casein* genotype	Animals with both 50K SNP and *α*_*s1*_ *casein* genotype
Alpine	Males	512	2154	510
	Females	1237	1542	0
Saanen	Males	393	2049	393
	Females	813	1457	0

### Genomic prediction with and without considering information on the ***α***_***s*1**_*casein* genotypes

ssGBLUP was implemented in 2017 in the official genetic evaluations for the two main French dairy goats. This method and pedigree-based BLUP were used as the reference method in our study and compared with WssGBLUP, two alternatives of the WssGBLUP method, TABLUP and the gene content method. Analyses were performed using the blupf90 software [38].

### Single-step GBLUP (ssGBLUP) method

For both multi-breed and within-breed scenarios, the following model was applied:

1 $${\mathbf{y}} = {\mathbf{X{\varvec{\upbeta}}}} + {\mathbf{Zu}} + {\mathbf{Wp}} + {\mathbf{e}},$$

where ${\mathbf{y}}$ is a vector of performances (female phenotypes) for protein content (phenotypes are based on standardized 250-day lactation records). ${\varvec{\upbeta}}$ is a vector of fixed effects including herd within year (32 years from 1980 to 2012) and within parity (1, 2 and ≥ 3) (188,933 levels in total); age at delivery within year and within region (four regions in France depending on goat breeding management) (3224 levels in total); month at delivery within year and region (1448 levels in total); and length of dry period within year and region (1107 levels in total); a fifth fixed effect for breed (two levels) was added for multi-breed analyses. ${\mathbf{u}}$ is a vector of random additive genetic effects assumed to be normally distributed $N\left( {0,{\mathbf{H}}\sigma_{u}^{2} } \right)$, ${\mathbf{p}}$ is a vector of random permanent environmental effects assumed to be normally distributed $N\left( {0,{\mathbf{I}}\sigma_{p}^{2} } \right)$, ${\mathbf{e}}$ is a vector of random residuals that is normally distributed $N\left( {0,{\mathbf{I}}\sigma_{e}^{2} } \right)$. ${\mathbf{X}}$ is the incidence matrix relating phenotypes to the fixed effects (${\varvec{\upbeta}}$**)**. ${\mathbf{Z}}$ is the design matrix allocating phenotypes to breeding values (${\mathbf{u}}$) and ${\mathbf{W}}$ is the incidence matrix relating phenotypes to permanent environmental effects (${\mathbf{p}}$).

Matrix ${\mathbf{H}}$ is the genetic relationship matrix combining SNP information and pedigree data, implemented as in Legarra et al. [12]:

$${\mathbf{H}} = \left( {\begin{array}{*{20}c} {{\mathbf{A}}_{11} + {\mathbf{A}}_{12} {\mathbf{A}}_{22}^{ - 1} \left( {{\mathbf{G}} - {\mathbf{A}}_{22} } \right){\mathbf{A}}_{22}^{ - 1} {\mathbf{A}}_{21} } & {{\mathbf{A}}_{12} {\mathbf{A}}_{22}^{ - 1} {\mathbf{G}}} \\ {{\mathbf{GA}}_{22}^{ - 1} {\mathbf{A}}_{21} } & {\mathbf{G}} \\ \end{array} } \right),$$

where ${\mathbf{A}}$ is a pedigree-based relationship matrix with indices 1 for ungenotyped animals and 2 for genotyped animals, and ${\mathbf{G}}$ is the genomic relationship matrix derived as in Christensen and Lund [11]:

$${\mathbf{G}} = 0.95\frac{{{\mathbf{M^{\prime}M}}}}{{2\mathop \sum \nolimits_{{{\text{i}} = 1}}^{\text{m}} p_{\text{i}} \left( {1 - p_{\text{i}} } \right)}} + 0.05{\mathbf{A}}_{22} ,$$

where ${\text{m}}$ is the number of SNPs, $p_{\text{i}}$ is the estimated allele frequency at locus $i$ and ${\mathbf{M}}$ is a centered matrix of SNP genotypes.

Variance components were estimated by using the restricted maximum likelihood (REML) method in the remlf90 software [38].

### Weighted ssGBLUP (WssGBLUP) method

Model 1 was also used for WssGBLUP but ${\mathbf{G}}$ was constructed differently. Solutions of genomic breeding values from ssGBLUP (Model 1) can be decomposed into SNP effects as modeled in Wang et al. [23]:

$${\hat{\mathbf{a}}} = {\mathbf{DM}}^{{\prime }} \left[ {{\mathbf{MDM}}^{{\prime }} } \right]^{ - 1} {\hat{\mathbf{u}}}_{g} ,$$

where **â** is a vector of SNP effects, **D** is a diagonal matrix of weights (initially diagonal of 1 for the ssGBLUP), ${\mathbf{M}}$ is the centered matrix of SNP genotypes and **û**_*g*_ the vector of GEBV from genotyped animals only. Variances of the effect of SNP $i$ were estimated as:

$$\sigma_{u,i}^{2} = 2\hat{a}_{i}^{2} p_{i} \left( {1 - p_{i} } \right),$$

where $p_{i}$ is the allele frequency of SNP $i$. The vector of variances of SNP effects was normalized (the normalization process ensured that the sum of the variances remained constant and was equal to the number of SNPs) and used as weights in matrix ${\mathbf{D}}$ to construct the weighted matrix ${\mathbf{G}}$ (${\mathbf{G}}^{ *}$) as described in Wang et al. [23]:

$${\mathbf{G}}^{ *} = 0.95\frac{{{\mathbf{M}}^{{\prime }} {\mathbf{DM}}}}{{2\mathop \sum \nolimits_{i = 1}^{{\mathbf{m}}} p_{i} \left( {1 - p_{i} } \right)}} + 0.05{\mathbf{A}}_{22} .$$

GEBV were estimated again with Model 1 by considering weights for each SNP via the ${\mathbf{G}}^{ *}$ matrix included in the ${\mathbf{H}}$ matrix. This process was carried out iteratively with weights estimated at each iteration as described in Wang et al. [23]. Wang et al. [23] have shown that WssGLUP with only very few iterations may be sufficient to reach a maximum accuracy of GEBV and SNP effects. In this study, we analyzed the influence of the number of iterations (1–10) on the accuracy of genomic predictions.

As proposed by Zhang et al. [24], other methods can be considered to calculate the weight for SNPs in the ${\mathbf{D}}$ matrix. These methods assign the same weight to several consecutive SNPs within a chromosomal region. Modifications of the WssGBLUP method were considered in this study and the individual weights were computed as follows: (1) the maximum weight of SNPs included in the chromosomal region, or (2) the sum of the weights of the SNPs included in the chromosomal region. These weights were calculated based on the weights estimated with the WssGBLUP. In the end, the vector of the weights was normalized in such a way that the sum of all weights remained constant and equal to the number of SNPs. Chromosomal regions of various lengths were tested: 2, 5, 10, 20, 40, 80, 100, 150, 200 and 250 consecutive SNPs with non-overlapping windows. Hereafter, these methods are named WssGBLUP_*i*_ where *i* denotes the method used to calculate the weights (Max or Sum).

### Trait-specific marker-derived relationship matrix (TABLUP) method

Only a subset of SNPs that are more or less associated with protein content was selected to build the ${\mathbf{G}}$ matrix. One of our objectives was to investigate how the genetic architecture of protein content could be taken into account in the ssGBLUP method. Thus, TABLUP was applied by selecting a subset of SNPs according to their effect on the trait (estimated from the WssGBLUP method described previously). A total of 5000, 10,000, 15,000, 20,000, 25,000, 30,000, 35,000 or 40,000 SNPs were selected to construct ${\mathbf{G}}$. Two scenarios were tested in which either the most or the least strongly associated SNPs were selected. GEBV were estimated with Model 1 and the ${\mathbf{G}}$ matrix that was built based on the selected SNPs without weights (${\mathbf{D}} = {\mathbf{I}}$).

### Gene content method

The gene content method estimates the GEBV for each animal by taking information on the *α*_*s1*_ *casein* genotype, genotypes from the 50K SNP and pedigree into account through a multiple-trait model. The model used here was the same as in [33]:

2 $$\begin{aligned} &{{\mathbf{y}} = {\mathbf{X{\varvec{\upbeta}}}} + {\mathbf{Zu}} + {\mathbf{Wp}} + {\mathbf{e}}} \hfill \\ &{ {\mathbf{y}}_{{A}} = {\mu }_{{A}} + {\mathbf{Z}}_{{A}} {\mathbf{u}}_{{A}} + {\mathbf{e}}_{{A}} } \hfill \\ &{{\mathbf{y}}_{{B}} = {\mu }_{{B}} + {\mathbf{Z}}_{{B}} {\mathbf{u}}_{{B}} + {\mathbf{e}}_{{B}} } \\ &{{\mathbf{y}}_{{C}} = {\mu }_{{C}} + {\mathbf{Z}}_{C} {\mathbf{u}}_{{C}} + {\mathbf{e}}_{{C}} } \\ &{ {\mathbf{y}}_{{E}} = {\mu }_{{E}} + {\mathbf{Z}}_{{E}} {\mathbf{u}}_{{E}} + {\mathbf{e}}_{{E}} } \\ &{ {\mathbf{y}}_{{F}} = {\mu }_{{F}} + {\mathbf{Z}}_{{F}} {\mathbf{u}}_{{F}} + {\mathbf{e}}_{{F}} } \\ &{ {\mathbf{y}}_{{O}} = {\mu }_{{O}} + {\mathbf{Z}}_{{O}} {\mathbf{u}}_{{O}} + {\mathbf{e}}_{{O}}} \\ \end{aligned},$$

where ${\mathbf{y}}$ is a vector of female performances for protein content. Fixed effects (${\varvec{\upbeta}}$), random effects (${\mathbf{u}}$, ${\mathbf{p}}$ and ${\mathbf{e}}$) and incidence matrices ${\mathbf{X}}$, ${\mathbf{Z}}$ and ${\mathbf{W}}$ are the same as in Model 1. ${\mathbf{y}}_{A}$, ${\mathbf{y}}_{B}$, ${\mathbf{y}}_{C}$, ${\mathbf{y}}_{E}$, ${\mathbf{y}}_{F}$, and ${\mathbf{y}}_{O}$ are vectors of gene content for alleles $A$, $B$, $C$, $E$, $F$ and $O$. This corresponds to the number of copies carried by each animal (i.e., 0, 1 or 2). For ungenotyped animals, the value was set to missing. ${\upmu}_{A}$, ${\upmu}_{B}$, ${\upmu}_{C}$, ${\upmu}_{E}$, ${\upmu}_{F}$, and ${\upmu}_{O}$ are the mean fixed effects for alleles $A$, $B$, $C$, $E$, $F$ and $O$, ${\mathbf{Z}}_{A}$, ${\mathbf{Z}}_{B}$, ${\mathbf{Z}}_{C}$, ${\mathbf{Z}}_{E}$, ${\mathbf{Z}}_{F}$, and ${\mathbf{Z}}_{O}$ are the incidence matrices relating observations to the random genetic effect (${\mathbf{u}}_{A}$, ${\mathbf{u}}_{B}$, ${\mathbf{u}}_{C}$, ${\mathbf{u}}_{E}$, ${\mathbf{u}}_{F}$ and ${\mathbf{u}}_{O}$) of gene content for each allele and ${\mathbf{e}}_{A}$, ${\mathbf{e}}_{B}$, ${\mathbf{e}}_{C}$, ${\mathbf{e}}_{E}$ and ${\mathbf{e}}_{O}$ are the random residual errors for each of the six alleles. For $i \in \left\{ {A, B, C, E, F, O} \right\}$, ${\mathbf{u}}_{\text{i}}$ are normally distributed such that $Var \left( {{\mathbf{u}}_{\text{i}} } \right) = {\mathbf{H}}\sigma_{{u_{\text{i}} }}^{2}$ and $\sigma_{{u_{i} }}^{2} = 2p_{i} \left( {1 - p_{i} } \right)$, where $p_{i}$ is the frequency of allele $i$ at the *α*_*s1*_ *casein* locus. Covariances between genetic values (${\mathbf{u}}$) and genetic effects of gene content (${\mathbf{u}}_{A}$, ${\mathbf{u}}_{B}$, ${\mathbf{u}}_{C}$, ${\mathbf{u}}_{E}$, ${\mathbf{u}}_{F}$ and ${\mathbf{u}}_{O}$) were modeled as in Carillier-Jacquin et al. [33]. Variance and covariance parameters from this model were estimated using the restricted maximum likelihood (REML) algorithm implemented in the remlf90 software.

### Accuracy of genomic predictions

Genomic evaluations were performed from all phenotypes recorded until January 2010, but we were also interested in the prediction of genotyped animals that constituted our reference population. This reference population was composed of 905 sires born between 1993 and 2012 and genotyped with the 50K SNP chip (Table 2) and was split into a training population of 554 sires born from 1993 to 2007 (307 Alpine and 247 Saanen) with phenotypes of their daughters recorded until January 2010), and 351 validation sires born from 2008 to 2012 (205 Alpine and 146 Saanen) with no daughters in January 2013 (daughters of these animals were removed from the dataset). Then, GEBV and DYD computed from the official genetic evaluation of January 2016 were compared for the 351 animals in the validation set. DYD were average performance values for the daughters corrected for environmental effects and merit of the dam, and they were weighted by effective daughter contributions as described in VanRaden and Wiggans [39]. Accuracy of genomic predictions was assessed as the Pearson correlation between GEBV estimated with each model and DYD. Pearson correlations obtained with different methods were tested using the Hotelling-Williams test [40].

## Results and discussion

The most frequent *α*_*s1*_ *casein* genotypes are $AA$ for the males and $AE$ for the females in the Alpine breed, and $AE$ for the females and $EE$ for the males in the Saanen breed (present in more than 50% of the animals). Allele $C$ is rather rare (less than 5% of the animals carry this allele) in the two breeds. The largest differences in genotype frequency between Alpine and Saanen populations were observed for genotypes $AA$ (49% in Alpine vs. 7% in Saanen), $EE$ (3% in Alpine vs. 32% in Saanen) and $AE$ (49% in Saanen *vs*. 30% in Alpine). These results were consistent with the previous work of Carillier-Jacquin et al. [33] in which fewer genotypes were available. Protein content was analyzed knowing that this trait is highly heritable in both Alpine and Saanen populations (0.5) [41].

### Estimation of weights for SNPs with the WssGBLUP method

**Fig. 1:**
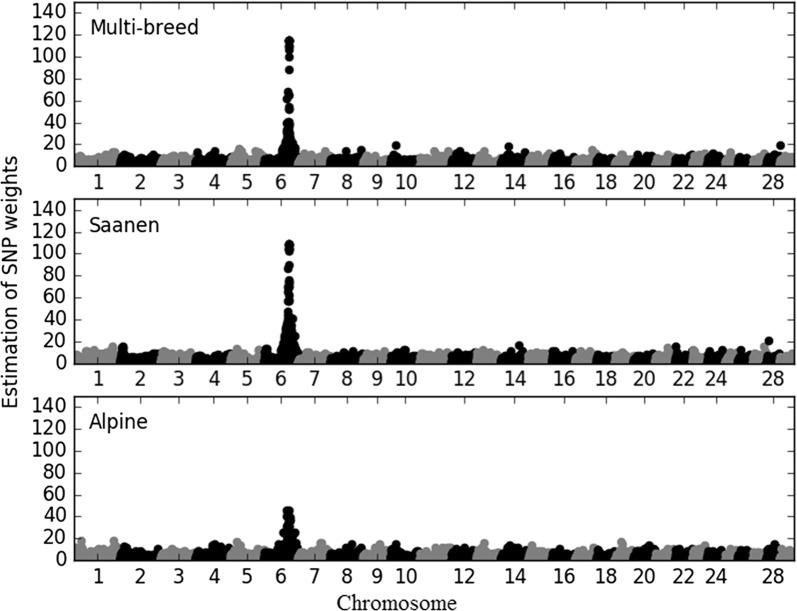
Estimated weights of SNPs included in the second iteration of the WssGBLUP approach for multi-breed, Alpine and Saanen populations. WssGBLUP used GEBV of genotyped animals and genotypes to estimate weights for each SNP. The estimated weight of each SNP is represented along the genome

We compared different genomic methods. First, we used WssGBLUP because we wanted to identify the weights given to SNPs with this method, in order to determine if the chromosomal region including the *α*_*s1*_ *casein* gene was considered in the analyses. WssGBLUP is an iterative method, and 10 iterations were performed for multi-breed analyses and within-breed analyses. Accuracy of genomic predictions was evaluated at each iteration (results not shown). The highest accuracies were obtained at the second iteration as reported by Wang et al. [23] and then decreased slightly. Thus, all the results presented for the WssGBLUP multi-breed and within-breed analyses are those obtained for the second iteration (see Fig. 1). The top 50 SNPs (with the biggest weights) were compared between the three analyses and were all located on chromosome 6 i.e. the multi-breed (between 71 and 86 Mb), Alpine (between 64 and 101 Mb) and Saanen analyses (between 71 and 92 Mb), and their weights ranged from 24 to 115 for multi-breed, from 23 to 45 for Alpine and from 30 to 108 for Saanen analyses. Among these SNPs, 16 were common to the three analyses and located between 78 and 82 Mb; 11 SNPs were common to the Saanen and multi-breed analyses and located between 79 and 83 Mb; 16 SNPs were common to the Alpine and multi-breed analyses and located between 77 and 86 Mb; and only one SNP was common to both the Alpine and Saanen analyses and located at 76 Mb.

WssGBLUP can be used not only for genomic prediction but also for QTL detection as in GWAS [23, 24]. In French dairy goat data, the chromosomal regions detected with WssGBLUP were on caprine chromosome 6, which includes a well-known region that was previously located and described by Martin et al. [34] in a GWAS study. They performed linkage analyses (LA) and linkage disequilibrium (LD) analyses on 1941 dairy goats distributed in 20 half-sib families using all females and their 20 sire genotypes and detected a large QTL between 82.5 and 82.8 Mb on chromosome 6. In our study, SNPs with the biggest weights for SNP variances were located within this region.

The WssGBLUP method developed by Wang et al. [23] has some limitations. Weights for SNP variances are estimated by using a whole-genome regression, which can result in their unstable prediction due to multi-collinearity between SNPs because of LD between SNPs. In our study, we tested common weights for several SNPs instead of individual weights for SNP variances, using WssGBLUP_Max_ or WssGBLUP_Sum_. These methods are expected to limit the large variation in prediction of weights for SNP variances by smoothing weights of SNPs that are in the same window. In our study, WssGBLUP_Max_ and WssGBLUP_Sum_ gave higher accuracies of genomic prediction than the classical WssGBLUP. With WssGBLUP_Max_ or WssGBLUP_Sum_, window sizes were used to allocate the same weights to consecutive SNPs. Another approach would be to use the LD between SNPs, which could limit the multi-collinearity between the SNPs used in the genomic evaluation. Since the weight of SNPs is included through the **D** matrix, this matrix can be replaced by the weights derived from the GWAS approach.

### Including the effect of the *α*_*S1*_*casein* gene in WssGBLUP or gene content methods

**Fig. 2:**
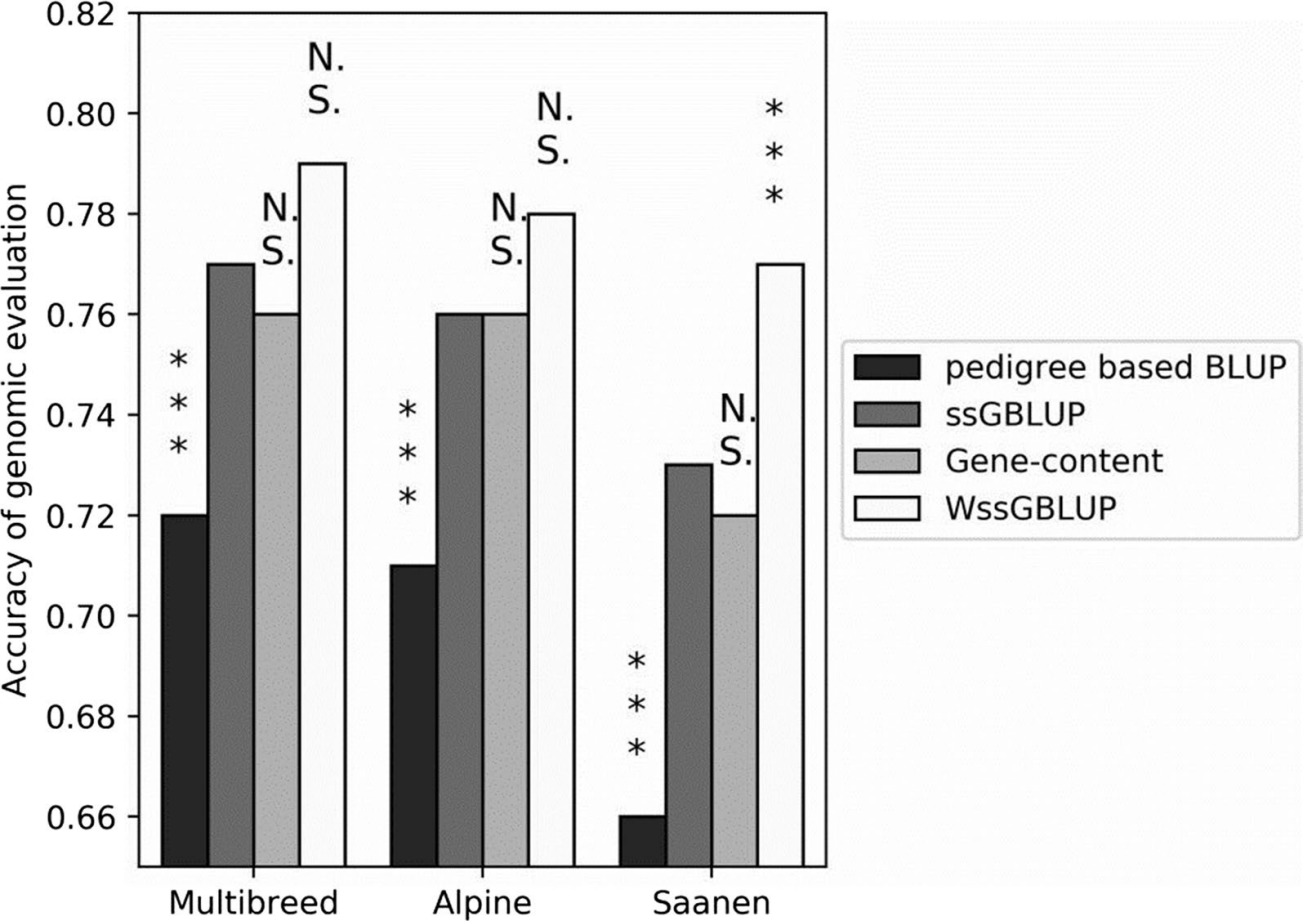
Validation correlations for validation males in multi-breed, Alpine and Saanen populations for pedigree-based BLUP, ssGBLUP, gene content and WssGBLUP approaches. Differences in accuracy between ssGBLUP and other approaches were tested with the Hotelling-Williams test (threshold: *5%, **3%, ***1%, *NS* non-significant)

Figure 2 presents accuracies of genomic evaluation for pedigree-based BLUP, ssGBLUP, gene content and WssGBLUP in a multi-breed population and in the Alpine and Saanen breeds. Accuracies with pedigree-based BLUP (0.72 in multi-breed, 0.71 in Alpine and 0.66 in Saanen) were lower than accuracies with ssGBLUP (0.77 in multi-breed, 0.76 in Alpine and 0.73 in Saanen), gene content (0.76 in multi-breed, 0.76 in Alpine and 0.72 in Saanen) or WssGBLUP (0.79 for multi-breed, 0.78 for Alpine and 0.77 for Saanen). The gene content method did not improve accuracy of genomic predictions for the three populations compared to ssGBLUP (accuracy was 1 percent point lower for gene content in the multi-breed and Saanen analyses and identical in the Alpine analysis). In addition, accuracies with WssGBLUP were significantly higher than with ssGBLUP for the Saanen population (+ 4 percent points). We did not observe any significant difference between ssGBLUP and WssGBLUP for multi-breed and Alpine populations.

Previously, Carillier-Jacquin et al. [33] used the gene content and ssGBLUP methods to analyze protein content in French dairy goats. Accuracies obtained with ssGBLUP were higher in our study than in Carillier-Jacquin et al. [33] for the multi-breed (+ 5 percent points) and Alpine (+ 8 percent points) analyses, and slightly lower for the Saanen analysis (− 2 percent points). A similar trend was observed with the gene content method, with + 1 percent point for multi-breed, + 8 percent points for Alpine and − 14 percent points for Saanen in our study compared to Carillier-Jacquin et al. [33]. The main difference between our study and that of Carillier-Jacquin et al. [33] was the number of animals genotyped with the 50 K SNP chip, number of *α*_*s1*_ *casein* genotypes, and the size and composition of the training and validation sets. In our study, 82 males and 2050 females genotyped with the 50 K SNP chip and 50 females and 878 males genotyped for the *α*_*s1*_ *casein* gene were added. In Carillier-Jacquin et al. [33], the reference population consisted of a training set with 677 animals born between 1993 and 2009 (384 Alpine and 293 Saanen), and a validation set with 146 animals born between 2010 and 2011 (86 Alpine and 60 Saanen). In our study, we had 554 animals born between 1993 and 2007 (307 Alpine and 247 Saanen) in the training set and 351 animals born between 2008 and 2012 (205 Alpine and 146 Saanen) in the validation set. The main difference between the Carillier-Jacquin et al. study and that reported here was the size of the validation population (2 versus 5 years in our study). The slightly improved results that we obtained may be explained by the larger reference population (823 animals in Carillier-Jacquin et al. [33] compared to 905 in our study), a well-known factor in the literature on genomic selection. For instance, VanRaden et al. [42] report a gain of + 5 percent points between genomic prediction and parent average by adding 1000 animals in the training population. These results were consistent with the higher accuracy obtained in the multi-breed analysis compared to the within-breed analyses, especially if the trait has the same genetic determinism in the two breeds that are combined (which is the case for protein content). Accuracy is expected to improve even more the size of the reference population continues to grow over the years.

Carillier-Jacquin et al. [33] showed that the gene content method was more accurate than ssGBLUP (+ 3 percent points for multi-breed, + 5 percent points for Alpine and + 11 percent points for Saanen). However, in our study, accuracies of genomic prediction were the same for the gene content method and ssGBLUP. The goat *α*_*s1*_ *casein* gene has six alleles in the two main French dairy goats and genotype frequencies vary considerably with some being rare. Predicting *α*_*s1*_ *casein* genotypes with the gene content method for non-genotyped animals remains difficult in this case, especially in French dairy goats, for which the number of non-genotyped animals is large compared with that of genotyped animals (only 0.3% of the population is genotyped for the *α*_*s1*_ *casein* gene), and 40% of females have unknown parents. This may explain why the gene content method did not outperform ssGBLUP.

The genetic architecture of protein content is similar between the Alpine and Saanen breeds. However, the gain in accuracy with the genomic evaluation methods (ssGBLUP, gene content and WssGBLUP) compared to pedigree-based BLUP was greater for the Saanen than the Alpine breed. As discussed by Carillier-Jacquin et al. [9], the greater gain observed for the Saanen breed between pedigree-based BLUP and genomic evaluation may be explained by a higher level of inbreeding (2.3% in Saanen and 1.8% in Alpine), and a higher kinship coefficient between the training and validation sets (2.4% in Saanen and 1.1% in Alpine using genomic data).

For prediction of GEBV, WssGBLUP was more efficient than gene content, which may be due to the construction of the 50K SNP chip. The region around the *α*_*s1*_ *casein* gene was enriched in SNPs in the 1-Mb region at 82 Mb on chromosome 6 (the region that contains the *α*_*s1*_ *casein* gene). Overall, 40 SNPs are present within this 1-Mb region, whereas on average only 20 SNPs per Mb are located outside of this region on chromosome 6 or on other chromosomes. Moreover, the Chi squared test between *α*_*s1*_ *casein* genotypes and each SNP on chromosome 6 revealed a very strong correlation between *α*_*s1*_ *casein* genotypes and SNPs on the 50K SNP chip in this region (results not shown). Giving more weight to SNPs that are more strongly associated with protein content seems to be more efficient to capture the effect of the *α*_*s1*_ *casein* gene than using genotype data for this gene. Vallejo et al. [18] investigated the efficiency of WssGBLUP for bacterial cold water disease resistance, for which several QTL are identified. They observed an improvement of 4 percent points with WssGBLUP compared to ssGBLUP. In our study, we observed similar gains with WssGBLUP. Su et al. [43] also observed a superiority of the WssGBLUP over ssGBLUP in dairy cattle for milk traits.

### Use of common weights on consecutive SNPs with WssGBLUP

WssGBLUP was significantly more predictive than other genomic evaluation methods for protein content in the Saanen breed but not in multi-breed or the Alpine breed. Zhang et al. [24] reported that WssGBLUP_Max_ and WssGBLUP_Sum_ increase the accuracy of genomic evaluation more efficiently than WssGBLUP. We evaluated these methods and Tables 3, 4 and 5 show the results on the validation population in the multi-breed, Alpine and Saanen populations, respectively using WssGBLUP and the two modified WssGBLUP methods (Max, Sum) according to the size of SNP windows. If identical results were obtained for different window sizes, they were merged in the same column. For the multi-breed population, accuracies of the analyses with WssGBLUP_Max_ and WssGBLUP_Sum_ were very similar and differed only with non-overlapping SNP windows of 40, 80, 100, 150, 200 and 250 SNPs, the accuracy (0.81) of WssGBLUP_Sum_ being slightly higher than that of WssGBLUP_Max_ (0.80). Otherwise, accuracies were equal to 0.79 with a window size of two SNPs and 0.80 for window sizes of five, 10 and 20 SNPs. Finally, accuracies of WssGBLUP_Max_ and WssGBLUP_Sum_ were slightly higher than that of WssGBLUP (0.79) and higher than that of ssGBLUP (0.77).

**Table 3:** Validation correlations for 351 validation males in the multi-breed population using different WssGBLUP and different window sizes of non-overlapping SNPs

Method	Size of non-overlapping SNP windows			
	1	2	5/10/20	40/80/100/150/200/250
WssGBLUP^a^	0.79			
WSSGBLUP_Sum_		0.79	0.80	0.81
WSSGBLUP_Max_		0.79	0.80	0.80

^a^Each SNP has its own weight (WssGBLUP standard)

**Table 4:** Validation correlations for 205 validation males in the Alpine breed using different WssGBLUP and different window sizes of non-overlapping SNPs

Method	Size of non-overlapping SNP windows				
	1	2	5/10/20	40	80/100/150/200/250
WssGBLUP^a^	0.78				
WSSGBLUP_Sum_		0.78	0.78	0.79	0.78
WSSGBLUP_Max_		0.77	0.78	0.78	0.78

^a^Each SNP has its own weight (WssGBLUP standard)

**Table 5:** Validation correlations for 146 validation males in the Saanen breed using different WssGBLUP and different windows size of non-overlapping SNPs

Method	Size of non-overlapping SNP window				
	1	2/5/10/20	40	80/100	150/200/250
WssGBLUP^a^	0.77				
WSSGBLUP_Sum_		0.77	0.78	0.78	0.78
WSSGBLUP_Max_		0.77	0.77	0.78	0.77

^a^Each SNP has its own weight (WssGBLUP standard)

For both within-breed analyses, increasing the window size barely influenced accuracies. In the Alpine within-breed analysis, a maximum accuracy of 0.79 was reached with the WssGBLUP_Sum_ method and a window size of 40 SNPs and thus, it outperformed WssGBLUP (0.78). For other window sizes (larger or smaller), accuracies with WssGBLUP_Sum_ were equal to 0.78. With the WssGBLUP_Max_ method, accuracies ranged from 0.77 for a window of two consecutive SNPs to 0.78 for windows of 5, 10, 20, 40, 80, 100, 150, 200 and 250 consecutive SNPs. In comparison, genomic evaluations with WssGBLUP_Max_ and WssGBLUP_Sum_ were more accurate than with ssGBLUP (0.76). In the Saanen within-breed analysis, accuracies of 0.78 were reached with WssGBLUP_Sum_ for windows of 40, 80, 100, 150, 200 and 250 consecutive SNPs, and with WssGBLUP_Max_ for windows of 80 and 100 consecutive SNPs. WssGBLUP_Max_ and WssGBLUP_Sum_ outperformed WssGBLUP (0.77) or even ssGBLUP (0.73). Accuracies of 0.77 were obtained with WssGBLUP_Sum_ for windows of 2, 5, 10 and 20 consecutive SNPs and with WssGBLUP_Max_ for windows of 2, 5, 10, 20, 40, 150, 200 and 250 consecutive SNPs.

WssGBLUP_Max_ and WssGBLUP_Sum_ slightly improved the accuracy of genomic predictions for protein content in French dairy goats compared to WssGBLUP. Similar results were observed by Zhang et al. [24] with WssGBLUP_Max_ and WssGBLUP_Sum_ compared to WssGBLUP on simulated data for five QTL. Zhang et al. [27] presented their results for a window size of 20 consecutive SNPs because when they used windows with more than 20 SNPs, accuracies decreased when many QTL affected a trait. This is due to most of the weight being assigned to the windows with large SNP effects and less weight to those with small SNP effects, which may introduce bias in the estimates. For the populations in our study, accuracies varied little with window size. However, 20 consecutive SNPs were not sufficient to reach the highest accuracies and 40 consecutive SNPs were more appropriate. Thus, for a trait that is influenced by few QTL, WssGBLUP_Max_ or WssGBLUP_Sum_ were more efficient to capture clear signals from QTL compared to WssGBLUP with one weight per SNP.

### TABLUP method

**Fig. 3:**
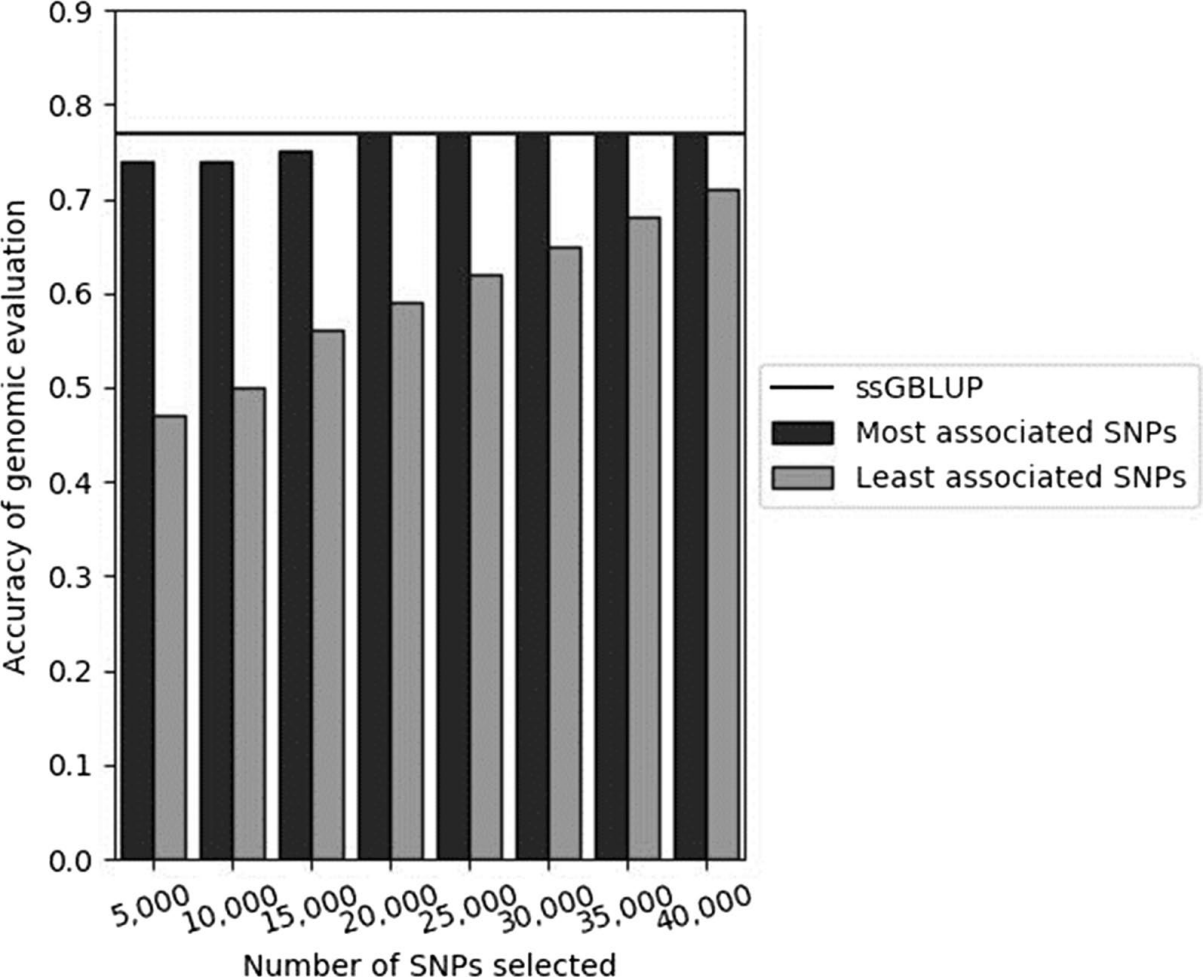
Validation correlations for 351 validation males in the multi-breed population using the TABLUP approach. TABLUP consists in selecting a subset of the genotypes from the 50K SNPs according to their association with protein content (either the most strongly associated or the least strongly associated with protein content), which is done by selecting SNPs according to their weights estimated with the WssGBLUP approach

To validate that ssGBLUP does capture the *α*_*s1*_ *casein* gene information, we used TABLUP that consists in selecting a subset of SNPs for constructing the ${\mathbf{G}}$ matrix, i.e. we selected the SNPs that were the most or the least strongly associated with protein content. Figure 3 shows the accuracies obtained with ssGBLUP and TABLUP for the multi-breed population according to the number of SNPs conserved (5000 to 40,000 SNPs) to construct the ${\mathbf{G}}$ matrix. Since results for both Alpine and Saanen breeds were similar to those for the multi-breed population, they are not shown.

First, for the SNPs that were the most strongly associated with protein content, TABLUP with only 5000 such SNPs led to a high accuracy of genomic prediction (0.74), which is close to that obtained with ssGBLUP (0.77). TABLUP reached the 0.77 accuracy of ssGBLUP with 20,000 such SNPs, which were distributed across the whole genome with on average 42% of the SNPs on each chromosome being retained and 54% on chromosome 6. This indicates that SNPs around the *α*_*s1*_ *casein* gene have been more selected than the others. Increasing the number of SNPs from 20,000 to 40,000, did not increase the accuracy furthermore. Conversely, for the SNPs that were the least strongly associated with protein content, TABLUP with 5000 such SNPs led to a very low accuracy (0.47) and increasing their number to 40,000 led to an increase in accuracy of 24 percent points (0.47 with 5000 SNPs and 0.71 with 40,000 SNPs) but accuracy remained significantly lower than that obtained by using the whole 50K SNP BeadChip (0.71 against 0.77).

Using different subsets of SNPs and the BayesA model, VanRaden et al. [44] compared accuracies of genomic predictions in Holstein breed cattle for 33 traits. They used 60K and high-density (HD) SNP panels, and added specific SNPs selected from whole-genome sequence data, which were SNPs based on their annotation (located on exons, splicing sites, indels, 2 kb upstream, 1 kb downstream, untranslated regions, SNPs with large effects). They showed that the highest accuracies were obtained with the scenario that used 60K SNPs plus the top 1000 SNPs for all 33 traits. Increasing the number of SNPs (using the HD SNP panel for example) did not increase the accuracy of genomic predictions. However, adding selected SNPs from whole-genome sequence to a medium-density SNP BeadChip improved GEBV accuracies. These results agree with those that we obtained with the TABLUP method. In the near future, when whole-genome caprine sequence data become available, it will be possible to select sequence-based variants and add them to the 50K SNP data in the genomic evaluation model, which will improve the accuracy of genomic predictions in these species.

We undertook additional analyses (results not shown) in which SNPs were removed chromosome-wise with the ssGBLUP, WssGBLUP and gene content methods. The same accuracies were observed, regardless of the chromosome from which the SNPs were removed, except for chromosome 6 for ssGBLUP (0.77), WssGBLUP (0.79) and gene content (0.76). When SNPs from chromosome 6 were removed, accuracies dropped to 0.70 for ssGBLUP, 0.66 for WssGBLUP and 0.74 for gene content. However, the loss in accuracy with gene content was smaller than with ssGBLUP and WssGBLUP, i.e. using genotypes for the *α*_*s1*_ *casein* gene and SNPs from 28 chromosomes (except chromosome 6) is quite similar to using the 50K SNP chip. The missing genotypes from the 50K SNP chip (i.e. the SNPs on chromosome 6) did not add much information compared to the information contained by the genotypes for the *α*_*s1*_ *casein*. Results of TABLUP and chromosome-wise removal of SNPs showed that a part of the effect of the *α*_*s1*_ *casein* gene was retained by the ssGBLUP method, which basically does not include information on causal mutations. These results can be explained by the high coverage of SNPs on chromosome 6 around the *α*_*s1*_ *casein* gene.

## Conclusions

Our aim was to investigate different genomic evaluation methods (using *α*_*s1*_ *casein* genotypes and/or 50K SNP information) to integrate information on the *α*_*s1*_ *casein* gene in genomic evaluations of dairy goats. Using the trait-specific marker-derived relationship matrix did not improve accuracy of genomic evaluation, which was the same as that obtained by ssGBLUP with a selection of the 20,000 most important SNPs for protein content. With the gene content method, accuracies of genomic evaluation were not improved compared to ssGBLUP, which is probably due to the *α*_*s1*_ *casein* gene having many alleles and to the small number of genotyped animals. Putting more weight on SNPs with larger effects improved accuracies of genomic evaluation using WssGBLUP, WssGBLUP_Max_ and WssGBLUP_Sum_. For WssGBLUP_Max_ and WssGBLUP_Sum_, accuracies were highest when a common weight was applied to non-overlapping windows of 40 SNPs. Gains in accuracies reached + 12 percent points for the Saanen, + 9 percent points for the multi-breed and + 8 percent points for the Alpine populations compared to a pedigree-based BLUP evaluation. WssGBLUP using common weights for SNPs within non-overlapping windows is efficient if the trait is influenced by few QTL and the true number of QTL is not known. WssGBLUP also combines fast computing and simplicity, and requires ssGBLUP to be run only twice.
